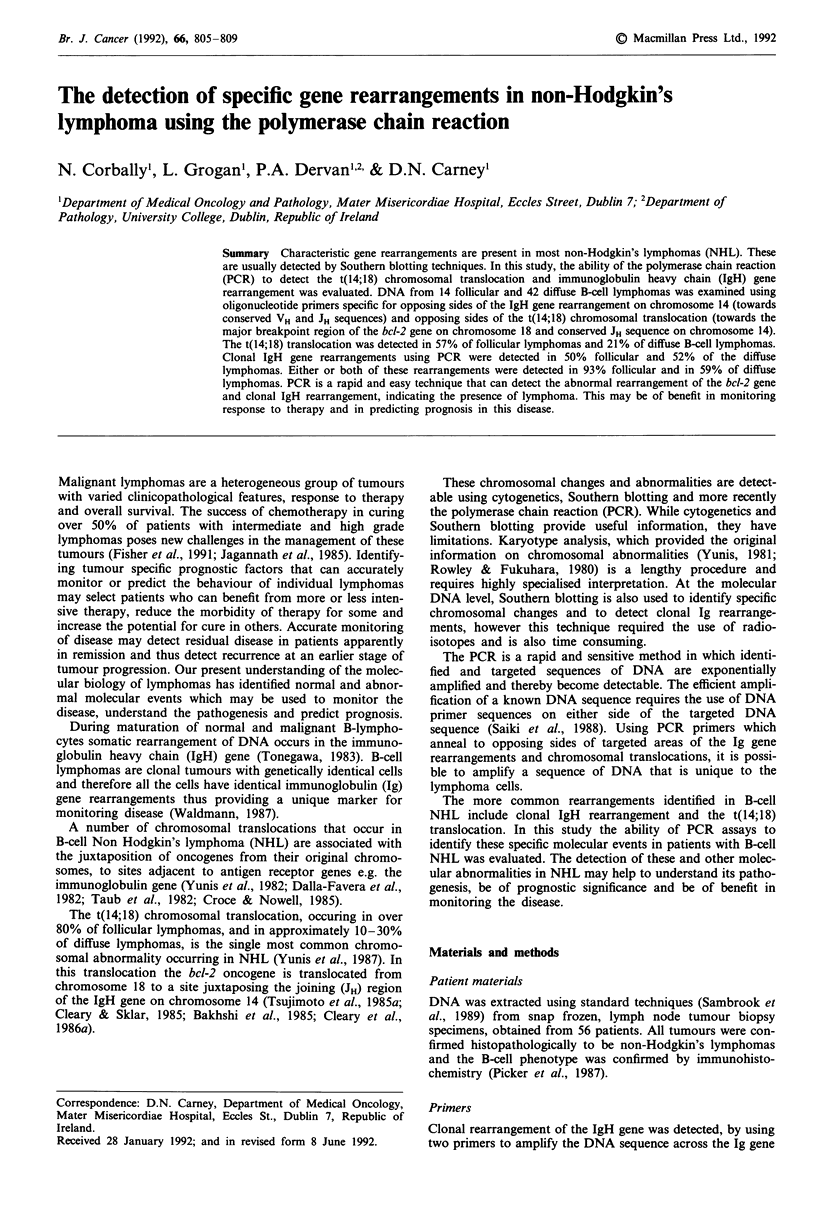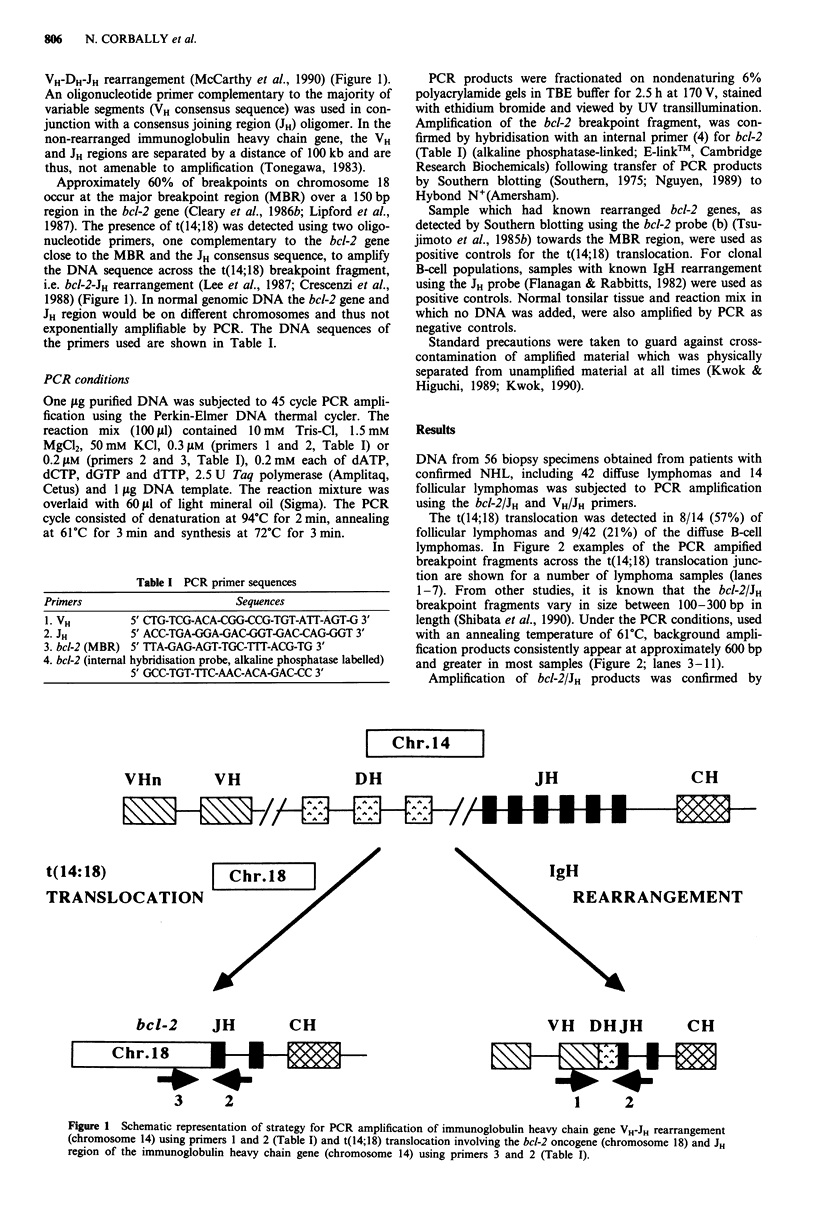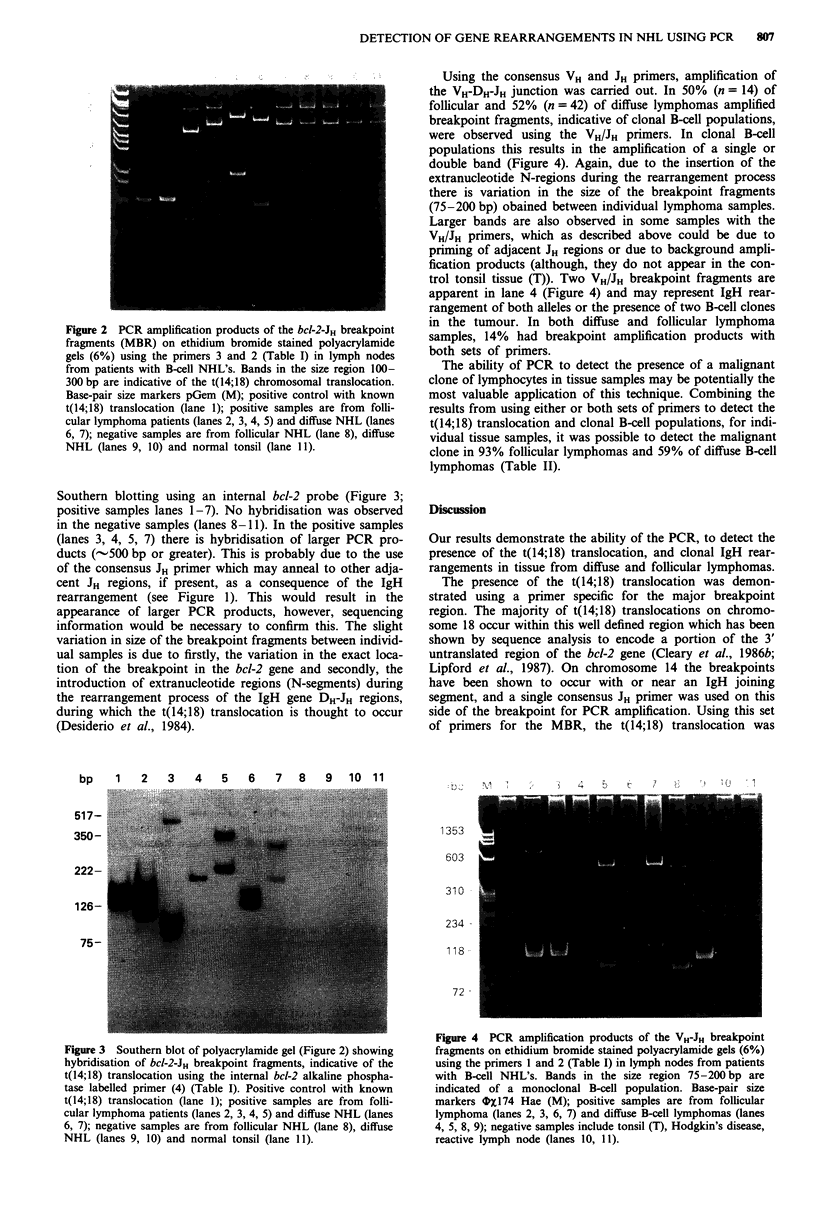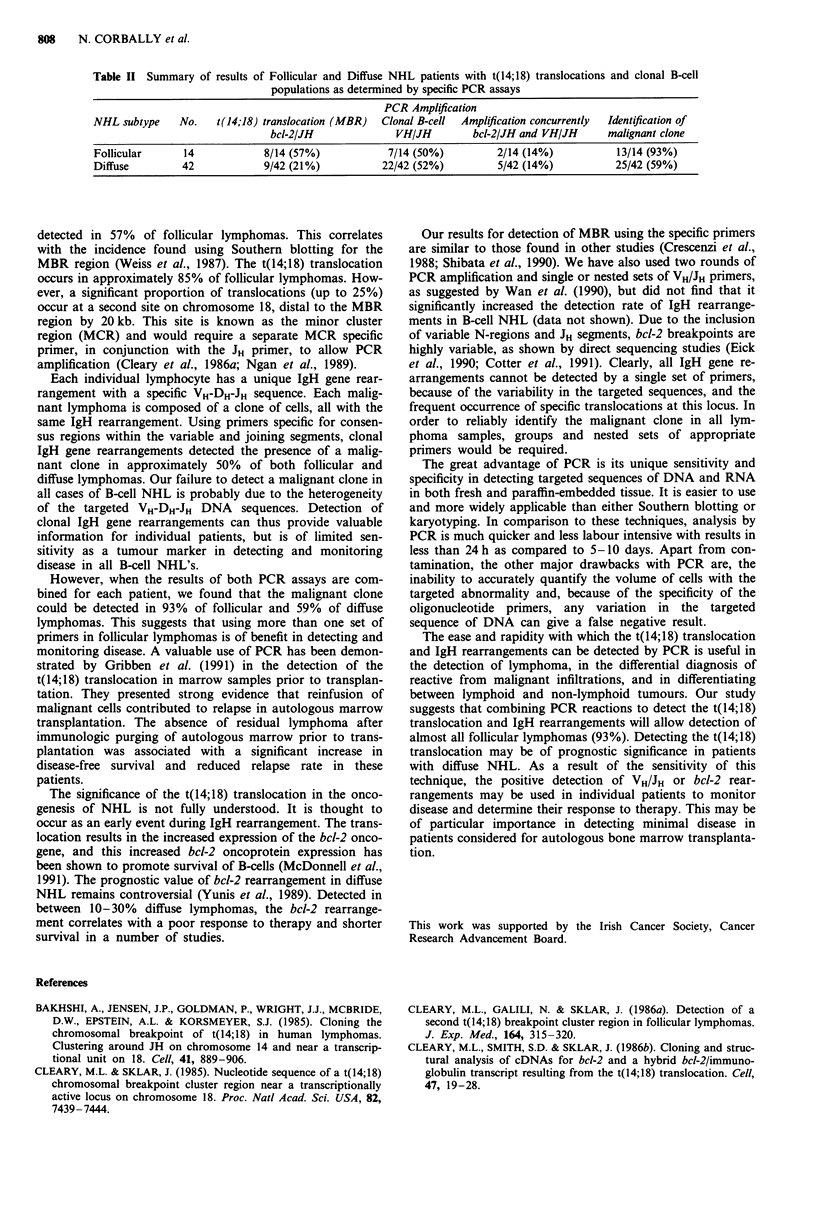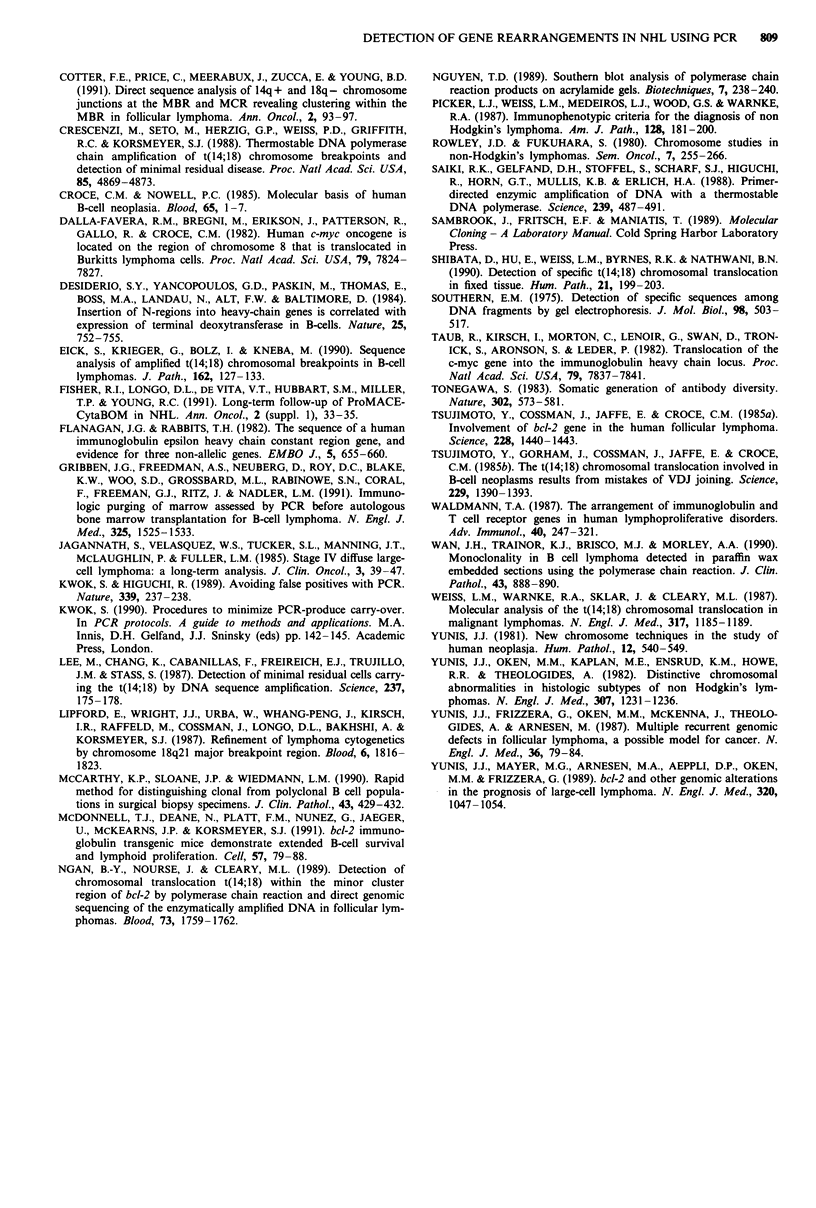# The detection of specific gene rearrangements in non-Hodgkin's lymphoma using the polymerase chain reaction.

**DOI:** 10.1038/bjc.1992.364

**Published:** 1992-11

**Authors:** N. Corbally, L. Grogan, P. A. Dervan, D. N. Carney

**Affiliations:** Department of Medical Oncology and Pathology, Mater Misericordiae Hospital, Dublin, Republic of Ireland.

## Abstract

**Images:**


*Br. J. Cancer* (1992), **66**, 805–809 © Macmillan Press Ltd., 1992

### The detection of specific gene rearrangements in non-Hodgkin's lymphoma using the polymerase chain reaction

N. Corbally[1], L. Grogan[1], P.A. Dervan[1,2] & D.N. Carney[1]

[1]*Department of Medical Oncology and Pathology, Mater Misericordiae Hospital, Eccles Street, Dublin 7;* [2]*Department of Pathology, University College, Dublin, Republic of Ireland*

**Summary** Characteristic gene rearrangements are present in most non-Hodgkin's lymphomas (NHL). These are usually detected by Southern blotting techniques. In this study, the ability of the polymerase chain reaction (PCR) to detect the t(14;18) chromosomal translocation and immunoglobulin heavy chain (IgH) gene rearrangement was evaluated. DNA from 14 follicular and 42 diffuse B-cell lymphomas was examined using oligonucleotide primers specific for opposing sides of the IgH gene rearrangement on chromosome 14 (towards conserved $V_H$ and $J_H$ sequences) and opposing sides of the t(14;18) chromosomal translocation (towards the major breakpoint region of the *bcl-2* gene on chromosome 18 and conserved $J_H$ sequence on chromosome 14). The t(14;18) translocation was detected in 57% of follicular lymphomas and 21% of diffuse B-cell lymphomas. Clonal IgH gene rearrangements using PCR were detected in 50% follicular and 52% of the diffuse lymphomas. Either or both of these rearrangements were detected in 93% follicular and in 59% of diffuse lymphomas. PCR is a rapid and easy technique that can detect the abnormal rearrangement of the *bcl-2* gene and clonal IgH rearrangement, indicating the presence of lymphoma. This may be of benefit in monitoring response to therapy and in predicting prognosis in this disease.

Malignant lymphomas are a heterogeneous group of tumours with varied clinicopathological features, response to therapy and overall survival. The success of chemotherapy in curing over 50% of patients with intermediate and high grade lymphomas poses new challenges in the management of these tumours (Fisher *et al.*, 1991; Jagannath *et al.*, 1985). Identifying tumour specific prognostic factors that can accurately monitor or predict the behaviour of individual lymphomas may select patients who can benefit from more or less intensive therapy, reduce the morbidity of therapy for some and increase the potential for cure in others. Accurate monitoring of disease may detect residual disease in patients apparently in remission and thus detect recurrence at an earlier stage of tumour progression. Our present understanding of the molecular biology of lymphomas has identified normal and abnormal molecular events which may be used to monitor the disease, understand the pathogenesis and predict prognosis.

During maturation of normal and malignant B-lymphocytes somatic rearrangement of DNA occurs in the immunoglobulin heavy chain (IgH) gene (Tonegawa, 1983). B-cell lymphomas are clonal tumours with genetically identical cells and therefore all the cells have identical immunoglobulin (Ig) gene rearrangements thus providing a unique marker for monitoring disease (Waldmann, 1987).

A number of chromosomal translocations that occur in B-cell Non Hodgkin's lymphoma (NHL) are associated with the juxtaposition of oncogenes from their original chromosomes, to sites adjacent to antigen receptor genes e.g. the immunoglobulin gene (Yunis *et al.*, 1982; Dalla-Favera *et al.*, 1982; Taub *et al.*, 1982; Croce & Nowell, 1985).

The t(14;18) chromosomal translocation, occuring in over 80% of follicular lymphomas, and in approximately 10–30% of diffuse lymphomas, is the single most common chromosomal abnormality occurring in NHL (Yunis *et al.*, 1987). In this translocation the *bcl-2* oncogene is translocated from chromosome 18 to a site juxtaposing the joining ($J_H$) region of the IgH gene on chromosome 14 (Tsujimoto *et al.*, 1985*a*; Cleary & Sklar, 1985; Bakhshi *et al.*, 1985; Cleary *et al.*, 1986*a*).

These chromosomal changes and abnormalities are detectable using cytogenetics, Southern blotting and more recently the polymerase chain reaction (PCR). While cytogenetics and Southern blotting provide useful information, they have limitations. Karyotype analysis, which provided the original information on chromosomal abnormalities (Yunis, 1981; Rowley & Fukuhara, 1980) is a lengthy procedure and requires highly specialised interpretation. At the molecular DNA level, Southern blotting is also used to identify specific chromosomal changes and to detect clonal Ig rearrangements, however this technique required the use of radioisotopes and is also time consuming.

The PCR is a rapid and sensitive method in which identified and targeted sequences of DNA are exponentially amplified and thereby become detectable. The efficient amplification of a known DNA sequence requires the use of DNA primer sequences on either side of the targeted DNA sequence (Saiki *et al.*, 1988). Using PCR primers which anneal to opposing sides of targeted areas of the Ig gene rearrangements and chromosomal translocations, it is possible to amplify a sequence of DNA that is unique to the lymphoma cells.

The more common rearrangements identified in B-cell NHL include clonal IgH rearrangement and the t(14;18) translocation. In this study the ability of PCR assays to identify these specific molecular events in patients with B-cell NHL was evaluated. The detection of these and other molecular abnormalities in NHL may help to understand its pathogenesis, be of prognostic significance and be of benefit in monitoring the disease.

### Materials and methods

### *Patient materials*

DNA was extracted using standard techniques (Sambrook *et al.*, 1989) from snap frozen, lymph node tumour biopsy specimens, obtained from 56 patients. All tumours were confirmed histopathologically to be non-Hodgkin's lymphomas and the B-cell phenotype was confirmed by immunohistochemistry (Picker *et al.*, 1987).

### *Primers*

Clonal rearrangement of the IgH gene was detected, by using two primers to amplify the DNA sequence across the Ig gene

Correspondence: D.N. Carney, Department of Medical Oncology, Mater Misericordiae Hospital, Eccles St., Dublin 7, Republic of Ireland.
Received 28 January 1992; and in revised form 8 June 1992.

$V_H$-$D_H$-$J_H$ rearrangement (McCarthy et al., 1990) (Figure 1). An oligonucleotide primer complementary to the majority of variable segments ($V_H$ consensus sequence) was used in conjunction with a consensus joining region ($J_H$) oligomer. In the non-rearranged immunoglobulin heavy chain gene, the $V_H$ and $J_H$ regions are separated by a distance of 100 kb and are thus, not amenable to amplification (Tonegawa, 1983).

Approximately 60% of breakpoints on chromosome 18 occur at the major breakpoint region (MBR) over a 150 bp region in the *bcl-2* gene (Cleary et al., 1986b; Lipford et al., 1987). The presence of t(14;18) was detected using two oligonucleotide primers, one complementary to the *bcl-2* gene close to the MBR and the $J_H$ consensus sequence, to amplify the DNA sequence across the t(14;18) breakpoint fragment, i.e. *bcl-2*-$J_H$ rearrangement (Lee et al., 1987; Crescenzi et al., 1988) (Figure 1). In normal genomic DNA the *bcl-2* gene and $J_H$ region would be on different chromosomes and thus not exponentially amplifiable by PCR. The DNA sequences of the primers used are shown in Table I.

### *PCR conditions*

One μg purified DNA was subjected to 45 cycle PCR amplification using the Perkin-Elmer DNA thermal cycler. The reaction mix (100 μl) contained 10 mM Tris-Cl, 1.5 mM $MgCl_2$, 50 mM KCl, 0.3 μM (primers 1 and 2, Table I) or 0.2 μM (primers 2 and 3, Table I), 0.2 mM each of dATP, dCTP, dGTP and dTTP, 2.5 U *Taq* polymerase (Amplitaq, Cetus) and 1 μg DNA template. The reaction mixture was overlaid with 60 μl of light mineral oil (Sigma). The PCR cycle consisted of denaturation at 94°C for 2 min, annealing at 61°C for 3 min and synthesis at 72°C for 3 min.

**Table I** PCR primer sequences

| *Primers* | *Sequences* |
|---|---|
| 1. $V_H$ | 5′ CTG-TCG-ACA-CGG-CCG-TGT-ATT-AGT-G 3′ |
| 2. $J_H$ | 5′ ACC-TGA-GGA-GAC-GGT-GAC-CAG-GGT 3′ |
| 3. *bcl-2* (MBR) | 5′ TTA-GAG-AGT-TGC-TTT-ACG-TG 3′ |
| 4. *bcl-2* (internal | hybridisation probe, alkaline phosphatase labelled) |
| | 5′ GCC-TGT-TTC-AAC-ACA-GAC-CC 3′ |

PCR products were fractionated on nondenaturing 6% polyacrylamide gels in TBE buffer for 2.5 h at 170 V, stained with ethidium bromide and viewed by UV transillumination. Amplification of the *bcl-2* breakpoint fragment, was confirmed by hybridisation with an internal primer (4) for *bcl-2* (Table I) (alkaline phosphatase-linked; E-link™, Cambridge Research Biochemicals) following transfer of PCR products by Southern blotting (Southern, 1975; Nguyen, 1989) to Hybond N+(Amersham).

Sample which had known rearranged *bcl-2* genes, as detected by Southern blotting using the *bcl-2* probe (b) (Tsujimoto et al., 1985b) towards the MBR region, were used as positive controls for the t(14;18) translocation. For clonal B-cell populations, samples with known IgH rearrangement using the $J_H$ probe (Flanagan & Rabbitts, 1982) were used as positive controls. Normal tonsilar tissue and reaction mix in which no DNA was added, were also amplified by PCR as negative controls.

Standard precautions were taken to guard against cross-contamination of amplified material which was physically separated from unamplified material at all times (Kwok & Higuchi, 1989; Kwok, 1990).

### Results

DNA from 56 biopsy specimens obtained from patients with confirmed NHL, including 42 diffuse lymphomas and 14 follicular lymphomas was subjected to PCR amplification using the *bcl-2*/$J_H$ and $V_H$/$J_H$ primers.

The t(14;18) translocation was detected in 8/14 (57%) of follicular lymphomas and 9/42 (21%) of the diffuse B-cell lymphomas. In Figure 2 examples of the PCR ampified breakpoint fragments across the t(14;18) translocation junction are shown for a number of lymphoma samples (lanes 1–7). From other studies, it is known that the *bcl-2*/$J_H$ breakpoint fragments vary in size between 100–300 bp in length (Shibata et al., 1990). Under the PCR conditions, used with an annealing temperature of 61°C, background amplification products consistently appear at approximately 600 bp and greater in most samples (Figure 2; lanes 3–11).

Amplification of *bcl-2*/$J_H$ products was confirmed by

**Figure 1** Schematic representation of strategy for PCR amplification of immunoglobulin heavy chain gene $V_H$-$J_H$ rearrangement (chromosome 14) using primers 1 and 2 (Table I) and t(14;18) translocation involving the *bcl-2* oncogene (chromosome 18) and $J_H$ region of the immunoglobulin heavy chain gene (chromosome 14) using primers 3 and 2 (Table I).

**Figure 2** PCR amplification products of the *bcl-2*-$J_H$ breakpoint fragments (MBR) on ethidium bromide stained polyacrylamide gels (6%) using the primers 3 and 2 (Table I) in lymph nodes from patients with B-cell NHL's. Bands in the size region 100–300 bp are indicative of the t(14;18) chromosomal translocation. Base-pair size markers pGem (M); positive control with known t(14;18) translocation (lane 1); positive samples are from follicular lymphoma patients (lanes 2, 3, 4, 5) and diffuse NHL (lanes 6, 7); negative samples are from follicular NHL (lane 8), diffuse NHL (lanes 9, 10) and normal tonsil (lane 11).

Southern blotting using an internal *bcl-2* probe (Figure 3; positive samples lanes 1–7). No hybridisation was observed in the negative samples (lanes 8–11). In the positive samples (lanes 3, 4, 5, 7) there is hybridisation of larger PCR products (~500 bp or greater). This is probably due to the use of the consensus $J_H$ primer which may anneal to other adjacent $J_H$ regions, if present, as a consequence of the IgH rearrangement (see Figure 1). This would result in the appearance of larger PCR products, however, sequencing information would be necessary to confirm this. The slight variation in size of the breakpoint fragments between individual samples is due to firstly, the variation in the exact location of the breakpoint in the *bcl-2* gene and secondly, the introduction of extranucleotide regions (N-segments) during the rearrangement process of the IgH gene $D_H$-$J_H$ regions, during which the t(14;18) translocation is thought to occur (Desiderio *et al.*, 1984).

Using the consensus $V_H$ and $J_H$ primers, amplification of the $V_H$-$D_H$-$J_H$ junction was carried out. In 50% ($n = 14$) of follicular and 52% ($n = 42$) of diffuse lymphomas amplified breakpoint fragments, indicative of clonal B-cell populations, were observed using the $V_H/J_H$ primers. In clonal B-cell populations this results in the amplification of a single or double band (Figure 4). Again, due to the insertion of the extranucleotide N-regions during the rearrangement process there is variation in the size of the breakpoint fragments (75–200 bp) obained between individual lymphoma samples. Larger bands are also observed in some samples with the $V_H/J_H$ primers, which as described above could be due to priming of adjacent $J_H$ regions or due to background amplification products (although, they do not appear in the control tonsil tissue (T)). Two $V_H/J_H$ breakpoint fragments are apparent in lane 4 (Figure 4) and may represent IgH rearrangement of both alleles or the presence of two B-cell clones in the tumour. In both diffuse and follicular lymphoma samples, 14% had breakpoint amplification products with both sets of primers.

The ability of PCR to detect the presence of a malignant clone of lymphocytes in tissue samples may be potentially the most valuable application of this technique. Combining the results from using either or both sets of primers to detect the t(14;18) translocation and clonal B-cell populations, for individual tissue samples, it was possible to detect the malignant clone in 93% follicular lymphomas and 59% of diffuse B-cell lymphomas (Table II).

### Discussion

Our results demonstrate the ability of the PCR, to detect the presence of the t(14;18) translocation, and clonal IgH rearrangements in tissue from diffuse and follicular lymphomas.

The presence of the t(14;18) translocation was demonstrated using a primer specific for the major breakpoint region. The majority of t(14;18) translocations on chromosome 18 occur within this well defined region which has been shown by sequence analysis to encode a portion of the 3′ untranslated region of the *bcl-2* gene (Cleary *et al.*, 1986*b*; Lipford *et al.*, 1987). On chromosome 14 the breakpoints have been shown to occur with or near an IgH joining segment, and a single consensus $J_H$ primer was used on this side of the breakpoint for PCR amplification. Using this set of primers for the MBR, the t(14;18) translocation was

**Figure 3** Southern blot of polyacrylamide gel (Figure 2) showing hybridisation of *bcl-2*-$J_H$ breakpoint fragments, indicative of the t(14;18) translocation using the internal *bcl-2* alkaline phosphatase labelled primer (4) (Table I). Positive control with known t(14;18) translocation (lane 1); positive samples are from follicular lymphoma patients (lanes 2, 3, 4, 5) and diffuse NHL (lanes 6, 7); negative samples are from follicular NHL (lane 8), diffuse NHL (lanes 9, 10) and normal tonsil (lane 11).

**Figure 4** PCR amplification products of the $V_H$-$J_H$ breakpoint fragments on ethidium bromide stained polyacrylamide gels (6%) using the primers 1 and 2 (Table I) in lymph nodes from patients with B-cell NHL's. Bands in the size region 75–200 bp are indicated of a monoclonal B-cell population. Base-pair size markers $\Phi\chi174$ Hae (M); positive samples are from follicular lymphoma (lanes 2, 3, 6, 7) and diffuse B-cell lymphomas (lanes 4, 5, 8, 9); negative samples include tonsil (T), Hodgkin's disease, reactive lymph node (lanes 10, 11).

**Table II** Summary of results of Follicular and Diffuse NHL patients with t(14;18) translocations and clonal B-cell populations as determined by specific PCR assays

| *NHL subtype* | *No.* | *PCR Amplification* *t(14;18) translocation (MBR) bcl-2/JH* | *Clonal B-cell VH/JH* | *Amplification concurrently bcl-2/JH and VH/JH* | *Identification of malignant clone* |
|---|---|---|---|---|---|
| Follicular | 14 | 8/14 (57%) | 7/14 (50%) | 2/14 (14%) | 13/14 (93%) |
| Diffuse | 42 | 9/42 (21%) | 22/42 (52%) | 5/42 (14%) | 25/42 (59%) |

detected in 57% of follicular lymphomas. This correlates with the incidence found using Southern blotting for the MBR region (Weiss *et al.*, 1987). The t(14;18) translocation occurs in approximately 85% of follicular lymphomas. However, a significant proportion of translocations (up to 25%) occur at a second site on chromosome 18, distal to the MBR region by 20 kb. This site is known as the minor cluster region (MCR) and would require a separate MCR specific primer, in conjunction with the $J_H$ primer, to allow PCR amplification (Cleary *et al.*, 1986*a*; Ngan *et al.*, 1989).

Each individual lymphocyte has a unique IgH gene rearrangement with a specific $V_H$-$D_H$-$J_H$ sequence. Each malignant lymphoma is composed of a clone of cells, all with the same IgH rearrangement. Using primers specific for consensus regions within the variable and joining segments, clonal IgH gene rearrangements detected the presence of a malignant clone in approximately 50% of both follicular and diffuse lymphomas. Our failure to detect a malignant clone in all cases of B-cell NHL is probably due to the heterogeneity of the targeted $V_H$-$D_H$-$J_H$ DNA sequences. Detection of clonal IgH gene rearrangements can thus provide valuable information for individual patients, but is of limited sensitivity as a tumour marker in detecting and monitoring disease in all B-cell NHL's.

However, when the results of both PCR assays are combined for each patient, we found that the malignant clone could be detected in 93% of follicular and 59% of diffuse lymphomas. This suggests that using more than one set of primers in follicular lymphomas is of benefit in detecting and monitoring disease. A valuable use of PCR has been demonstrated by Gribben *et al.* (1991) in the detection of the t(14;18) translocation in marrow samples prior to transplantation. They presented strong evidence that reinfusion of malignant cells contributed to relapse in autologous marrow transplantation. The absence of residual lymphoma after immunologic purging of autologous marrow prior to transplantation was associated with a significant increase in disease-free survival and reduced relapse rate in these patients.

The significance of the t(14;18) translocation in the oncogenesis of NHL is not fully understood. It is thought to occur as an early event during IgH rearrangement. The translocation results in the increased expression of the *bcl-2* oncogene, and this increased *bcl-2* oncoprotein expression has been shown to promote survival of B-cells (McDonnell *et al.*, 1991). The prognostic value of *bcl-2* rearrangement in diffuse NHL remains controversial (Yunis *et al.*, 1989). Detected in between 10–30% diffuse lymphomas, the *bcl-2* rearrangement correlates with a poor response to therapy and shorter survival in a number of studies.

Our results for detection of MBR using the specific primers are similar to those found in other studies (Crescenzi *et al.*, 1988; Shibata *et al.*, 1990). We have also used two rounds of PCR amplification and single or nested sets of $V_H/J_H$ primers, as suggested by Wan *et al.* (1990), but did not find that it significantly increased the detection rate of IgH rearrangements in B-cell NHL (data not shown). Due to the inclusion of variable N-regions and $J_H$ segments, *bcl-2* breakpoints are highly variable, as shown by direct sequencing studies (Eick *et al.*, 1990; Cotter *et al.*, 1991). Clearly, all IgH gene rearrangements cannot be detected by a single set of primers, because of the variability in the targeted sequences, and the frequent occurrence of specific translocations at this locus. In order to reliably identify the malignant clone in all lymphoma samples, groups and nested sets of appropriate primers would be required.

The great advantage of PCR is its unique sensitivity and specificity in detecting targeted sequences of DNA and RNA in both fresh and paraffin-embedded tissue. It is easier to use and more widely applicable than either Southern blotting or karyotyping. In comparison to these techniques, analysis by PCR is much quicker and less labour intensive with results in less than 24 h as compared to 5–10 days. Apart from contamination, the other major drawbacks with PCR are, the inability to accurately quantify the volume of cells with the targeted abnormality and, because of the specificity of the oligonucleotide primers, any variation in the targeted sequence of DNA can give a false negative result.

The ease and rapidity with which the t(14;18) translocation and IgH rearrangements can be detected by PCR is useful in the detection of lymphoma, in the differential diagnosis of reactive from malignant infiltrations, and in differentiating between lymphoid and non-lymphoid tumours. Our study suggests that combining PCR reactions to detect the t(14;18) translocation and IgH rearrangements will allow detection of almost all follicular lymphomas (93%). Detecting the t(14;18) translocation may be of prognostic significance in patients with diffuse NHL. As a result of the sensitivity of this technique, the positive detection of $V_H/J_H$ or *bcl-2* rearrangements may be used in individual patients to monitor disease and determine their response to therapy. This may be of particular importance in detecting minimal disease in patients considered for autologous bone marrow transplantation.

This work was supported by the Irish Cancer Society, Cancer Research Advancement Board.

### References

BAKHSHI, A., JENSEN, J.P., GOLDMAN, P., WRIGHT, J.J., MCBRIDE, D.W., EPSTEIN, A.L. & KORSMEYER, S.J. (1985). Cloning the chromosomal breakpoint of t(14;18) in human lymphomas. Clustering around JH on chromosome 14 and near a transcriptional unit on 18. *Cell*, **41**, 889–906.

CLEARY, M.L. & SKLAR, J. (1985). Nucleotide sequence of a t(14;18) chromosomal breakpoint cluster region near a transcriptionally active locus on chromosome 18. *Proc. Natl Acad. Sci. USA*, **82**, 7439–7444.

CLEARY, M.L., GALILI, N. & SKLAR, J. (1986*a*). Detection of a second t(14;18) breakpoint cluster region in follicular lymphomas. *J. Exp. Med.*, **164**, 315–320.

CLEARY, M.L., SMITH, S.D. & SKLAR, J. (1986*b*). Cloning and structural analysis of cDNAs for *bcl-2* and a hybrid *bcl-2*/immunoglobulin transcript resulting from the t(14;18) translocation. *Cell*, **47**, 19–28.

COTTER, F.E., PRICE, C., MEERABUX, J., ZUCCA, E. & YOUNG, B.D. (1991). Direct sequence analysis of 14q+ and 18q− chromosome junctions at the MBR and MCR revealing clustering within the MBR in follicular lymphoma. *Ann. Oncol.*, **2**, 93–97.

CRESCENZI, M., SETO, M., HERZIG, G.P., WEISS, P.D., GRIFFITH, R.C. & KORSMEYER, S.J. (1988). Thermostable DNA polymerase chain amplification of t(14;18) chromosome breakpoints and detection of minimal residual disease. *Proc. Natl Acad. Sci. USA*, **85**, 4869–4873.

CROCE, C.M. & NOWELL, P.C. (1985). Molecular basis of human B-cell neoplasia. *Blood*, **65**, 1–7.

DALLA-FAVERA, R.M., BREGNI, M., ERIKSON, J., PATTERSON, R., GALLO, R. & CROCE, C.M. (1982). Human *c-myc* oncogene is located on the region of chromosome 8 that is translocated in Burkitts lymphoma cells. *Proc. Natl Acad. Sci. USA*, **79**, 7824–7827.

DESIDERIO, S.Y., YANCOPOULOS, G.D., PASKIN, M., THOMAS, E., BOSS, M.A., LANDAU, N., ALT, F.W. & BALTIMORE, D. (1984). Insertion of N-regions into heavy-chain genes is correlated with expression of terminal deoxytransferase in B-cells. *Nature*, **25**, 752–755.

EICK, S., KRIEGER, G., BOLZ, I. & KNEBA, M. (1990). Sequence analysis of amplified t(14;18) chromosomal breakpoints in B-cell lymphomas. *J. Path.*, **162**, 127–133.

FISHER, R.I., LONGO, D.L., DE VITA, V.T., HUBBART, S.M., MILLER, T.P. & YOUNG, R.C. (1991). Long-term follow-up of ProMACE-CytaBOM in NHL. *Ann. Oncol.*, **2** (suppl. 1), 33–35.

FLANAGAN, J.G. & RABBITS, T.H. (1982). The sequence of a human immunoglobulin epsilon heavy chain constant region gene, and evidence for three non-allelic genes. *EMBO J.*, **5**, 655–660.

GRIBBEN, J.G., FREEDMAN, A.S., NEUBERG, D., ROY, D.C., BLAKE, K.W., WOO, S.D., GROSSBARD, M.L., RABINOWE, S.N., CORAL, F., FREEMAN, G.J., RITZ, J. & NADLER, L.M. (1991). Immunologic purging of marrow assessed by PCR before autologous bone marrow transplantation for B-cell lymphoma. *N. Engl. J. Med.*, **325**, 1525–1533.

JAGANNATH, S., VELASQUEZ, W.S., TUCKER, S.L., MANNING, J.T., MCLAUGHLIN, P. & FULLER, L.M. (1985). Stage IV diffuse large-cell lymphoma: a long-term analysis. *J. Clin. Oncol.*, **3**, 39–47.

KWOK, S. & HIGUCHI, R. (1989). Avoiding false positives with PCR. *Nature*, **339**, 237–238.

KWOK, S. (1990). Procedures to minimize PCR-produce carry-over. In *PCR protocols. A guide to methods and applications.* M.A. Innis, D.H. Gelfand, J.J. Sninsky (eds) pp. 142–145. Academic Press, London.

LEE, M., CHANG, K., CABANILLAS, F., FREIREICH, E.J., TRUJILLO, J.M. & STASS, S. (1987). Detection of minimal residual cells carrying the t(14;18) by DNA sequence amplification. *Science*, **237**, 175–178.

LIPFORD, E., WRIGHT, J.J., URBA, W., WHANG-PENG, J., KIRSCH, I.R., RAFFELD, M., COSSMAN, J., LONGO, D.L., BAKHSHI, A. & KORSMEYER, S.J. (1987). Refinement of lymphoma cytogenetics by chromosome 18q21 major breakpoint region. *Blood*, **6**, 1816–1823.

MCCARTHY, K.P., SLOANE, J.P. & WIEDMANN, L.M. (1990). Rapid method for distinguishing clonal from polyclonal B cell populations in surgical biopsy specimens. *J. Clin. Pathol.*, **43**, 429–432.

MCDONNELL, T.J., DEANE, N., PLATT, F.M., NUNEZ, G., JAEGER, U., MCKEARNS, J.P. & KORSMEYER, S.J. (1991). *bcl-2* immunoglobulin transgenic mice demonstrate extended B-cell survival and lymphoid proliferation. *Cell*, **57**, 79–88.

NGAN, B.-Y., NOURSE, J. & CLEARY, M.L. (1989). Detection of chromosomal translocation t(14;18) within the minor cluster region of *bcl-2* by polymerase chain reaction and direct genomic sequencing of the enzymatically amplified DNA in follicular lymphomas. *Blood*, **73**, 1759–1762.

NGUYEN, T.D. (1989). Southern blot analysis of polymerase chain reaction products on acrylamide gels. *Biotechniques*, **7**, 238–240.

PICKER, L.J., WEISS, L.M., MEDEIROS, L.J., WOOD, G.S. & WARNKE, R.A. (1987). Immunophenotypic criteria for the diagnosis of non Hodgkin's lymphoma. *Am. J. Path.*, **128**, 181–200.

ROWLEY, J.D. & FUKUHARA, S. (1980). Chromosome studies in non-Hodgkin's lymphomas. *Sem. Oncol.*, **7**, 255–266.

SAIKI, R.K., GELFAND, D.H., STOFFEL, S., SCHARF, S.J., HIGUCHI, R., HORN, G.T., MULLIS, K.B. & ERLICH, H.A. (1988). Primer-directed enzymic amplification of DNA with a thermostable DNA polymerase. *Science*, **239**, 487–491.

SAMBROOK, J., FRITSCH, E.F. & MANIATIS, T. (1989). *Molecular Cloning – A Laboratory Manual.* Cold Spring Harbor Laboratory Press.

SHIBATA, D., HU, E., WEISS, L.M., BYRNES, R.K. & NATHWANI, B.N. (1990). Detection of specific t(14;18) chromosomal translocation in fixed tissue. *Hum. Path.*, **21**, 199–203.

SOUTHERN, E.M. (1975). Detection of specific sequences among DNA fragments by gel electrophoresis. *J. Mol. Biol.*, **98**, 503–517.

TAUB, R., KIRSCH, I., MORTON, C., LENOIR, G., SWAN, D., TRONICK, S., ARONSON, S. & LEDER, P. (1982). Translocation of the c-myc gene into the immunoglobulin heavy chain locus. *Proc. Natl Acad. Sci. USA*, **79**, 7837–7841.

TONEGAWA, S. (1983). Somatic generation of antibody diversity. *Nature*, **302**, 573–581.

TSUJIMOTO, Y., COSSMAN, J., JAFFE, E. & CROCE, C.M. (1985*a*). Involvement of *bcl-2* gene in the human follicular lymphoma. *Science*, **228**, 1440–1443.

TSUJIMOTO, Y., GORHAM, J., COSSMAN, J., JAFFE, E. & CROCE, C.M. (1985*b*). The t(14;18) chromosomal translocation involved in B-cell neoplasms results from mistakes of VDJ joining. *Science*, **229**, 1390–1393.

WALDMANN, T.A. (1987). The arrangement of immunoglobulin and T cell receptor genes in human lymphoproliferative disorders. *Adv. Immunol.*, **40**, 247–321.

WAN, J.H., TRAINOR, K.J., BRISCO, M.J. & MORLEY, A.A. (1990). Monoclonality in B cell lymphoma detected in paraffin wax embedded sections using the polymerase chain reaction. *J. Clin. Pathol.*, **43**, 888–890.

WEISS, L.M., WARNKE, R.A., SKLAR, J. & CLEARY, M.L. (1987). Molecular analysis of the t(14;18) chromosomal translocation in malignant lymphomas. *N. Engl. J. Med.*, **317**, 1185–1189.

YUNIS, J.J. (1981). New chromosome techniques in the study of human neoplasia. *Hum. Pathol.*, **12**, 540–549.

YUNIS, J.J., OKEN, M.M., KAPLAN, M.E., ENSRUD, K.M., HOWE, R.R. & THEOLOGIDES, A. (1982). Distinctive chromosomal abnormalities in histologic subtypes of non Hodgkin's lymphomas. *N. Engl. J. Med.*, **307**, 1231–1236.

YUNIS, J.J., FRIZZERA, G., OKEN, M.M., MCKENNA, J., THEOLOGIDES, A. & ARNESEN, M. (1987). Multiple recurrent genomic defects in follicular lymphoma, a possible model for cancer. *N. Engl. J. Med.*, **36**, 79–84.

YUNIS, J.J., MAYER, M.G., ARNESEN, M.A., AEPPLI, D.P., OKEN, M.M. & FRIZZERA, G. (1989). *bcl-2* and other genomic alterations in the prognosis of large-cell lymphoma. *N. Engl. J. Med.*, **320**, 1047–1054.